# Veterans Health Administration response to 2021 recall of Philips Respironics devices: A case study

**DOI:** 10.3389/frsle.2023.1129415

**Published:** 2023-04-12

**Authors:** Jeffrey K. Belkora, Barry Fields, Q. Afifa Shamim-Uzzaman, Donna Stratford, David Alfandre, Scott Hollingshaus, Edward Yackel, Cynthia Geppert, Penny Nechanicky, Ardene Nichols, Katherine Williams, Jill Reichert, Mary A. Whooley, Joe Francis, Kathleen F. Sarmiento

**Affiliations:** ^1^Institute for Health Policy Studies, University of California, San Francisco, San Francisco, CA, United States; ^2^San Francisco Veterans Affairs Health Care System, San Francisco, CA, United States; ^3^Division of Pulmonary, Allergy, Critical Care, and Sleep Medicine, Emory University, Atlanta, GA, United States; ^4^Atlanta Veterans Affairs Medical Center, Decatur, GA, United States; ^5^Department of Internal Medicine and Neurology, Veterans Affairs Ann Arbor Healthcare System, University of Michigan, Ann Arbor, MI, United States; ^6^Office of Quality and Patient Safety, Veterans Health Administration, Washington, DC, United States; ^7^Department of Medicine and Population Health, New York University School of Medicine, New York, NY, United States; ^8^National Center for Ethics in Health Care, Veterans Health Administration, Washington, DC, United States; ^9^Division of Pulmonary Medicine, Department of Medicine, Salt Lake City Veterans Affairs Medical Center, University of Utah, Salt Lake City, UT, United States; ^10^National Center for Patient Safety, Veterans Health Administration, Ann Arbor, MI, United States; ^11^Department of Psychiatry and Internal Medicine, Ethics Education, University of New Mexico School of Medicine, Albuquerque, NM, United States; ^12^Prosthetic and Sensory Aids Services, Veterans Health Administration, Washington, DC, United States; ^13^Department of Medicine, University of California, San Francisco, San Francisco, CA, United States; ^14^Office of Reporting Analytics, Performance, Improvement and Deployment, Veterans Health Administration, Washington, DC, United States

**Keywords:** sleep medicine, disordered breathing, positive airway pressure devices, ventilators, product safety, product recall, crisis, organizational behavior

## Abstract

This case study describes, for the time frame of June 2021 through August 2022, the U.S. Veterans Health Administration (VHA) organizational response to a manufacturer's recall of positive airway pressure devices used in the treatment of sleep disordered breathing. VHA estimated it could take over a year for Veterans to receive replacement devices. Veterans awaiting a replacement faced a dilemma. They could continue using the recalled devices and bear the product safety risks that led to the recall, or they could stop using them and bear the risks of untreated sleep disordered breathing. Using a program monitoring approach, we report on the processes VHA put in place to respond to the recall. Specifically, we report on the strategic, service, and operational plans associated with VHA's response to the recall for Veterans needing replacement devices. In program monitoring, the strategic plan reflects the internal process objectives for the program. The service plan articulates how the delivery of services will intersect the customer journey. The operational plan describes how the program's resources and actions must support the service delivery plan. VHA's strategic plan featured a clinician-led, as opposed to primarily legal or administrative response to the recall. The recall response team also engaged with VHA's medical ethics service to articulate an ethical framework guiding the allocation of replacement devices under conditions of scarcity. This framework proposed allocating scarce devices to Veterans according to their clinical need. The service plan invited Veterans to schedule visits with sleep providers who could assess their clinical need and counsel them accordingly. The operational plan distributed devices according to clinical need as they became available. Monitoring our program processes in real time helped VHA launch and adapt its response to a recall affecting more than 700,000 Veterans.

## 1. Introduction

### 1.1. Nature of problem being addressed

This case study addresses the worldwide recall of ~5 million positive airway pressure (PAP) and portable ventilator devices manufactured by Philips Respironics before April 26, 2021 (Philips Respironics, 2022a). Ventilators are used to help patients breathe or breathe for them. PAP devices are used to treat sleep-related breathing disorders such as obstructive sleep apnea (OSA), a condition in which the airway collapses during sleep. PAP ensures patients maintain an open airway and/or breathing rate. Disorders like OSA can lead to other health problems or even death if not treated adequately.

The background for the recall is that in 2020, ~1,300 consumers using particular Philips devices reported complaints to the manufacturer (Brauer et al., 2022). Eleven patients experienced unexpected symptoms that required treatment, including headache, upper airway irritation, cough, chest pressure, and sinus infection (Owens et al., 2021). There were no hospitalizations or deaths associated with the complaints. Several complaints reported the presence of black debris/particles within the airpath circuit extending from the device outlet, humidifier, tubing, and mask.

After launching an inquiry to understand the root cause of these complaints, Philips first reported the issue on April 26, 2021 in a Regulatory Update as part of its Quarterly Shareholder Report (Philips Respironics, 2021b) On June 14, 2021, Philips issued a voluntary recall (Philips Respironics, 2021c) of specific models of its CPAP devices, Bi-Level PAP Devices, and continuous ventilators (Trilogy 100, Trilogy 200, Garbin Plus, Aeris, LifeVent, BiPAP V30, and BiPAP A30/A40 Series Device Models). Philips Respironics found that the problems were more likely for older devices; those stored in conditions of high heat and humidity; and those exposed to ultraviolet light or ozone-based cleaning.

For patients using affected BiPAP or CPAP devices, Philips Respironics' initial guidance on their patient-facing website stated, “Stop use of bilevel PAP and CPAP sleep apnea devices.” (Philips Respironics, 2022b) After input from VHA and American Academy of Sleep Medicine, American Thoracic Society and other stakeholders (Owens et al., 2021), by November 16, 2021, Philips added a statement, “For patients using BiLevel PAP and CPAP devices, consult with your physician on a suitable treatment plan” (Philips Respironics, 2021a).

On June 30, 2021, the US Food and Drug Administration (FDA) designated this as a Class 1 recall (FDA, 2021), meaning “a situation in which there is a reasonable probability that the use of, or exposure to a violative product will cause serious adverse health consequences or death” (FDA, 2014). The manufacturer's proposed remedy to the FDA's designation of this as a class 1 recall was to have patients self-register their recalled device and replace each registered device on a first-come, first served basis. However, Philips did not have enough new devices on hand to replace all registered recalled devices at once; it would need to produce new devices and estimated that it would likely take at least a year to meet demand.

Many Veterans using recalled devices faced a dilemma while they waited for their replacement devices from Philips. On one hand, they could stop using recalled devices and bear the risks of untreated sleep disordered breathing. On the other hand, they could continue using recalled devices and bear the health risks that precipitated the recall.

In light of these risks, the Veterans Health Administration (VHA) marshaled an organizational response for the 725,145 Veterans who possessed a recalled device and relied on VHA for devices or supplies. This case study will focus on VHA's response to the recall for these Veterans.

This case study contributes to the emerging literature on the recall. Two reports provided background on the recall and guidance for how clinicians should respond (Owens et al., 2021; Shrivastava et al., 2021). Three reports elaborated on the health risks of recalled PAP devices, such as cancer (Kendzerska et al., 2021; Palm et al., 2022; Wang and Xiao, 2022). One report examined whether recalled PAP devices could be modified to reduce risks (Rabec et al., 2022). In our domain of organizational case studies, we found two accounts, one from Mayo Clinic (Morgenthaler et al., 2022) and one from the Cleveland Clinic (Lance et al., 2022).

The Cleveland Clinic recounts how it contacted 15,759 patients and disseminated a decision-making algorithm for providers to use in guiding these patients during visits and *via* smart-phrase messaging in the electronic health record and patient portal. Their report analyzes factors predicting which patients were advised to continue therapy with a recalled device while awaiting replacement. They found that patients advised to continue PAP therapy had “higher burden of sleep apnea and cardiopulmonary comorbidity.” The report does not provide a detailed account of the operations or logistics involved in responding to the recall, leaving a gap for others to address.

The Mayo Clinic addresses this gap by providing more details on their organization's operational response to the recall, which they estimate affected 9,000 patients. Like Cleveland Clinic, Mayo also created a decision algorithm, and crafted direct messages to patients. This report includes exhibits of the actual decision flowchart, and excerpts of the direct messages to patients. Mayo also recounts how it employed three lessons learned from prior recalls: ensure centralized awareness of the recall; help staff visualize a reasoned proactive approach; and use empathic communications to inform patients about the recall. This inductive framework, grounded in Mayo's experience, will be helpful to other organizations. However, the Mayo report does not use a deductive evaluation framework, leaving a gap for us to address.

Our experience complements these reports by adding to the literature the case of a large organization dealing with over 700,000 Veterans affected by the recall. To cope with the organizational complexity and volume, we employed an explicit framework for program monitoring in real time during the recall. Adding this framework and the details of our response to the reports from Cleveland Clinic and Mayo will allow other organizations to draw on similarities and differences in the experiences of diverse healthcare systems in the United States.

### 1.2. Rationale for proposed innovation

This case study describes an innovation in which VHA used a program monitoring framework to launch a clinician-led response to the recall, rather than leading with a legal, administrative, or logistical approach. The rationale for this innovation was that VHA clinicians were the ones who originally prescribed the recalled devices based on an assessment of relative benefits and harms. Clinicians were best positioned to revisit these decisions with Veterans. We describe key elements of the clinician-led response and hope that future recall responses may be informed by our experience.

## 2. Context

The setting for this case study was the Veterans Health Administration (VHA) healthcare system. VHA is America's largest integrated healthcare system, serving over 9 million enrolled Veterans each year at more than 170 medical centers and over 1,000 outpatient clinics (US Department of Veterans Affairs, 2021b). Within VHA, ~1.2 million Veterans have been diagnosed with obstructive sleep apnea in addition to other types of sleep disordered breathing (Folmer et al., 2020). VHA is the clinical provider as well as durable medical equipment distributor to these Veterans. This report focuses on VHA's efforts, during the period June 2021 through August 2022, to support Veterans under VHA care who were in possession of 725,145 recalled devices.

## 3. Detail to understand key programmatic elements

### 3.1. Monitoring the recall response

Any organizational response can be monitored in terms of its internal program processes (Rossi et al., 1999). Following Rossi and other evaluation scientists, we describe program processes in terms of the program's strategic plan; service plan; and operational plan. These correspond, respectively, to the high level process objectives for the program; the intended delivery of services along the customer journey; and to the organizational actions required to support the services delivered (Belkora et al., 2009). To use an everyday example, a casual dining restaurant might articulate a strategic plan stating it will serve healthy food made from fresh ingredients with minimal waiting time. The service plan might specify that customers will proceed cafeteria style down an assembly line comprised of fresh ingredients that servers will add to their plate. The operational plan might specify (among other things) which purveyors can hired to provide the best quality at the desired price; how the servers must be trained to answer questions about the menu; and how drinks will be positioned at the cash register at the end of the assembly line. The scope of this report is to describe program processes corresponding to VHA's strategic plan, service plan, and operational plan for the recall, as described below. Table 1 describes each process element and summarizes how our case example reflects that process. The sections of this report follow headings drawn from this table.

**Table 1:** Monitoring and reporting on program processes.

	**Program processes**		
	**Strategic plan**	**Service plan**	**Operational plan**
Description	High-level (strategic) process objectives	Customer or end-user journey	Organizational actions to support customer journey
Case example from VHA response to recall	i. Assemble a cross-functional recall response team ii. Defer to clinician assessment of Veteran clinical need iii. Communicate transparently about the recall iv. Allocate scarce resources based on an explicit ethical framework v. Invite Veterans to schedule appointments with VHA sleep providers (vs. relying on chart review)	i. Revisit initial decision about PAP therapy ii. Review potential benefits and harms of recalled devices (while waiting for replacement)	i. Identify Veterans affected by recall ii. Inform Veterans and invite them to schedule appointments with VHA sleep providers iii. Stratify Veterans by risk of harm iv. Help providers guide Veterans with a clinical note template

### 3.2. Strategic plan

The strategic plan summarizes the high level internal process objectives for a program. We articulated the following objectives for the recall program.

#### 3.2.1. Assembling a cross-functional recall response team

Organizationally, the recall fell under the purview of VHA's National Center for Patient Safety, which assembled a multi-disciplinary team including representatives from the National Center for Patient Safety; National Center for Ethics in Health Care; Prosthetics and Sensory Aids Services; Primary Care; Sleep Medicine; Clinical Episode Review Team; and Communications. This team met daily from 7/23/21; then Monday through Thursday until 3/3/22; and then Mondays and Thursdays after 3/3/22.

#### 3.2.2. Deferring to clinician assessment of Veteran clinical need

The recall response team had to react quickly to the decisions and announcements made by the manufacturer and by the U.S. Food and Drug Administration. The recall response team's strategy revolved around taking a Veteran-centered, clinician-led approach to the recall, in line with VHA's core values of Integrity, Commitment, Advocacy, Respect, and Excellence. The recall response team's orientation to defer to clinicians as leaders was also in line with VHA's commitment to the principles of being a high reliability organization, in this case the principle of deferring to expertise (Merchant et al., 2022).

The manufacturer initially advised consumers to discontinue using the affected devices until the manufacturer could replace the device. VHA clinicians joined others in raising concerns, given the risks of leaving untreated the sleep-related breathing disorders if consumers were to discontinue use of recalled devices as advised by the manufacturer (Owens et al., 2021).

#### 3.2.3. Communicating transparently about the recall

The recall response team embraced the strategic importance of Veteran and clinical communications. The team's composition included author DS, Director of Communication, from the VHA's Office of Quality and Patient Safety. Within a week after notification of the recall, VHA's communication team established a PAP Recall web page (US Department of Veterans Affairs, 2021a) and an internal administrative site for clinical and patient communications.

Starting in July 2021, the communication team notified all VHA public affairs officers about the recall and provided them with initial messages and responses to frequently asked questions (FAQ). The prosthetics team began gathering information on Veterans who either received a recalled device or ordered supplies for PAP devices. In addition, the communications team solicited from the Veterans Benefits Administration a list of Veterans receiving disability compensation for sleep apnea syndrome at a level that suggested use of a PAP device. The combined lists captured more than 1.2 million Veterans and served as the foundation for later emails and letters to patients (see operational plan below).

As the process of remediation evolved, the communications team facilitated the dissemination of information to both Veterans and clinicians in real time. For Veterans, the communications team mounted an email and postal mail campaign, as described in the operational plan below.

To reach clinicians, the communications team hosted virtual (online) office hours to educate clinicians on the recall. The office hours took place on an interactive webinar platform with an associated file sharing site and chat stream for participants to post questions either during the meeting or asynchronously throughout the week. The communications team invited 793 VHA sleep providers to the meeting series. Each session featured a presentation summarizing the recall processes to date and step-by-step instructions to implement the processes, and then opened the floor for questions and answers. In addition, the meetings were recorded and shared with attendees, along with files containing instructions and screenshots.

The recall response team also sent weekly to biweekly email updates to email distribution lists comprised of 1,145 sleep providers and other allied professionals, such as VA Prosthetics, Logistics, and supporting staff. Members of the team also monitored an email inbox set up to receive questions from the field.

In addition, the communications team set up a centralized repository of information within the National Center for Patient Safety SharePoint site. This is a document sharing platform accessible by employees. This repository included documents to keep clinicians up to date. It also included information for clinicians or public affairs or communication officers to share with Veterans. These documents included notifications about the recall; fact sheets; clinical decision guides; and frequently asked questions with answers (FAQs). The communications team updated these documents as the situation evolved. In addition, the communications team posted information about the recall on a public-facing website hosted by the National Center for Patient Safety (US Department of Veterans Affairs, 2021a; https://www.patientsafety.va.gov/safety-notice/philips-cpap-recall.asp); in VA social media channels; Veterans Benefits Administration and VA monthly bulletins; and VA's patient portal (HealtheVet). The goal was to be transparent in communications and build trust with Veterans.

Finally, the communications team hosted quarterly webinars to brief public affairs and communications officers around VHA on how to use the provided communications materials, such as email and letter templates, to guide Veterans at local facilities. The communications team provided VA Veterans Service Organization councils and committees with briefings, emails and communication materials to assist with outreach to their membership about the recall.

#### 3.2.4. Allocating scarce resources based on an explicit ethical framework

Anticipating a shortage of replacement devices from all manufacturers for 1–2 years, the recall response team identified the need for an ethical framework to guide allocation of the scarce replacement devices. VHA's National Center for Ethics in Health Care articulated the options: first come, first served; allocation according to clinical need; or lottery.

The recall response team adopted clinical need as the preferred approach to allocating scarce resources. VHA's National Center for Ethics in Health Care wrote a guidance document entitled “Meeting the Ethical Challenges of a Medical Device Shortage” (National Center for Ethics in Health Care, 2021).

Below are key excerpts summarizing the guidance:

During a global medical device shortage, the demand for treatment can outstrip the available supply which can affect the ability to adequately treat all patients…. VA's mission, values, and ethics principles obligate it to articulate and use a transparent ethical framework to ensure an equitable allocation process that promotes utility, that is, the greatest good for the greatest number of Veterans…. If all attempts to augment resources have been exhausted and there remain more patients with a clinical indication for treatment than there are available devices, a protocol for allocation of devices will be used. “Based on the principles of beneficence and utility, patients with a clinical indication for PAP who wish to receive a device should be stratified into a hierarchy for treatment.” This hierarchy is based on which patients are most likely to benefit from the treatment and which patients would be least harmed by delaying treatment…. Triage presumes that everyone with an indication for treatment will eventually be treated, but those who are less ill may wait longer.

#### 3.2.5. Inviting Veterans to schedule appointments with VHA sleep providers (vs. relying on chart review)

Once the recall response team had adopted clinical need as the basis for allocation, the next decision was how to implement the allocation. The team considered allocation based on chart review vs. allocation based on interaction with patients. The team concluded that chart review failed to take into consideration factors only obtainable through engagement with a patient. These included considerations such as use of ozone cleaners that increased the risk of foam degradation; visual observation of foam breakdown; and patient subjective appraisal of the benefits of pursuing PAP therapy with a replacement device vs. risks of continuing PAP therapy with a recalled device. Clinicians would need to know these inputs in order to follow the ethical allocation framework in determining patients “most likely to benefit from treatment.” Therefore, the team decided to implement the allocation framework by inviting patients to schedule appointments with sleep providers who could assess their individual experience and recommend treatment accordingly.

### 3.3. Service plan—Mapping the Veteran journey

In program monitoring, the service plan describes how a service provider's efforts will support the customer's journey. The VA's overall strategic plan also emphasizes the importance of beginning with the Veteran's journey in mind (US Department of Veterans Affairs, 2018). VA Strategy 1.1.3 states that “VA will expand the use of the Veteran journey maps to enhance our business functions, such as acting on operational risks that impact Veteran outcomes.”

**Figure 1:**
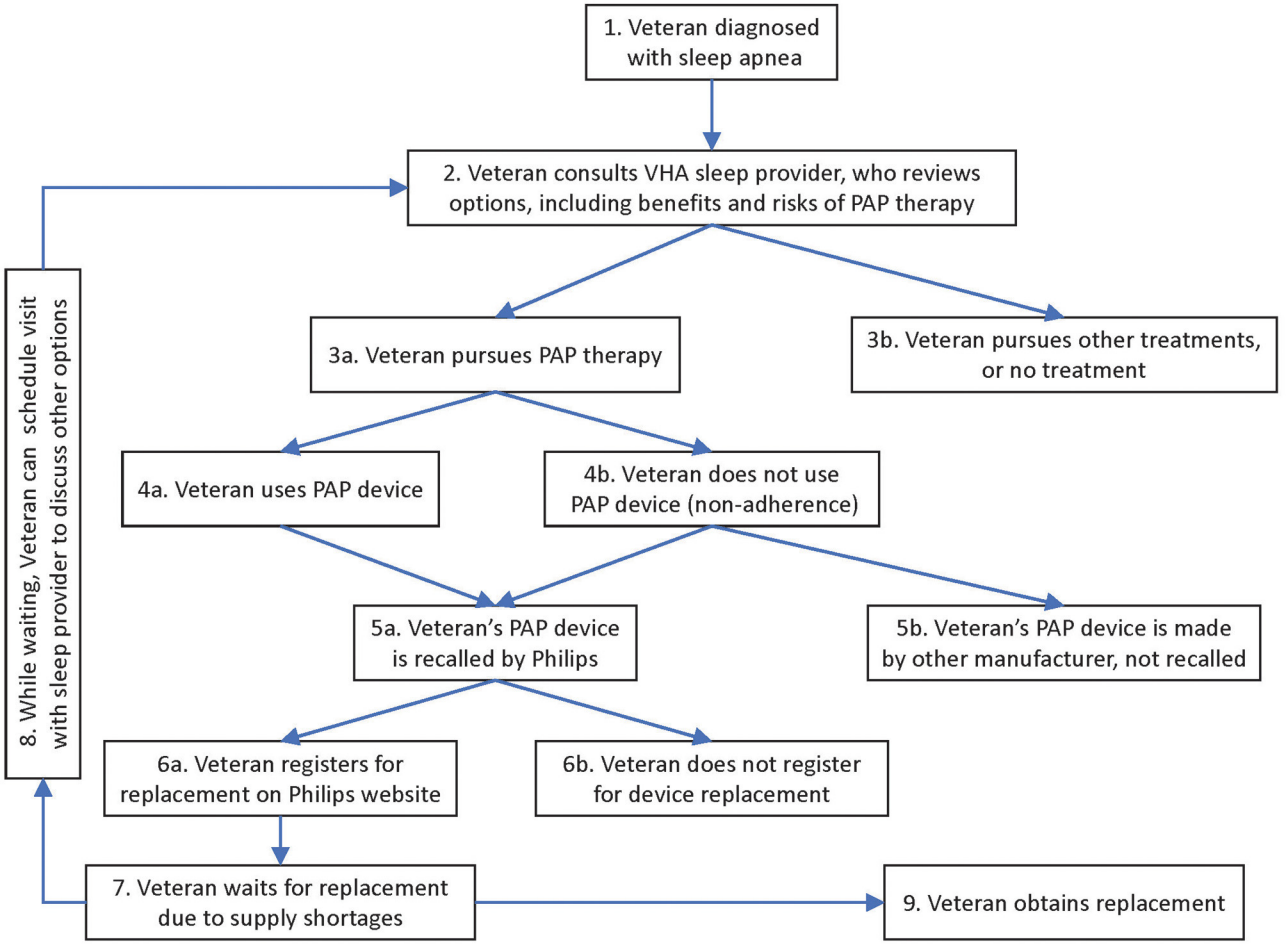
Patient (Veteran) journey.

We mapped the Veteran journey as follows (see Figure 1). In VHA, each Veteran's journey with PAP devices originally started with being diagnosed with sleep apnea (item 1 in Figure 1). The journey continued with the Veteran consulting a sleep provider, weighing the advantages and disadvantages of PAP devices relative to other options (2). Many Veterans pursued PAP therapy (3a), although some chose other treatments, or no treatment (3b). Veterans who received PAP devices either used them (4a) or were non-adherent (4b). These Veterans either had devices recalled by Philips (5a) or their devices were made by other manufacturers and not subject to recall (5b). Veterans with recalled devices either registered for their replacement (6a) or did not (6b). At this stage, while Veterans awaited a replacement device (7), the recall response team encouraged Veterans to schedule an appointment with their VHA specialist (returning to step 2, *via* step 8 in the diagram) to revisit the options. This time they evaluated how the risks and benefits of continuing PAP therapy with a recalled device compared to the risks and benefits of other therapies, including no treatment. Thus, Veterans would arrive at a temporary strategy (3a or 3b) while they waited for the replacement of their recalled device (9).

#### 3.3.1. Revisiting initial decision about PAP therapy

Our map of the Veteran journey stimulated the insight that the recall represented a revisiting of the original decision to adopt PAP therapy. The initial decision to prescribe a PAP device reflected the patient and provider's assessment that the benefits of the device outweighed the potential harms. The recall response team understood that some devices, under some conditions, might have degraded and now cause near-term symptoms, with a possible risk of longer-term harms.

In the recall response team's view, this meant that Veterans should work with their sleep providers to revisit their original decisions about using positive airway pressure devices. Ideally, Veterans would again weigh the benefits of using their device against the harms of using it—now in the context of the recall (which changed the risk/benefit profile); and in the context of Veterans having experience of using the devices.

#### 3.3.2. Reviewing potential benefits and harms of recalled PAP devices (while waiting for replacement)

Revisiting the initial decision required updating the Veteran's view of potential harms from PAP therapy with a recalled device, given 1,304 reports of consumers reporting complaints or symptoms. The manufacturer estimated the rate as ~0.03% of devices (Philips Respironics, 2021c). Beyond symptoms, the manufacturer was concerned that long-term harms might include life-threatening risks such as cancer.

Meanwhile, the benefits of continuing to use recalled devices while awaiting replacement could include reduced risk of accidents; cardiovascular events; daytime sleepiness; depression; stroke; substance abuse; and other negative outcomes associated with disordered breathing during sleep.

Therefore, the recall response team's prescription was to encourage Veterans to make their decisions about device usage in consultation with VHA providers. Supporting this service plan would require several large-scale initiatives comprising the recall response team's operational plan, described next.

### 3.4. Operational plan

In program monitoring, the operational plan describes the organizational actions and resources that must be deployed to support the service plan. Below we summarize VHA's key actions to support the service plan described above.

#### 3.4.1. Identifying Veterans affected by the recall

In order to invite affected Veterans to consult sleep providers about next steps, VHA needed to identify which Veterans were in possession of recalled devices. The recall response team reviewed a report generated by the National Prosthetic Patient Database, a database that contains Prosthetic and Sensory Aid Services transactional activity from each VHA facility. This report, along with facility-level analysis efforts, identified 502,358 Veterans issued Philips Respironics devices, and an additional list of Veterans who received supplies for recalled devices between 2009 and 2021, for a total of 725,145.

#### 3.4.2. Informing Veterans and inviting them to schedule appointments with VHA sleep providers

Having identified the target population, in August 2021 the communications team sent emails to the 725,145 Veterans identified as likely to be in possession of recalled devices. The open rate for these emails was 51% after three attempts, higher than the typical VA email open rate of 15%. In September 2021 the communications team provided the Government Printing Office with letters for 201,884 Veterans who were unreachable by email. These communications summarized the recall and the manufacturer's process for registering recalled devices for replacement, and pointed Veterans to a VA PAP Recall website with additional information and answers to Frequently Asked Questions (US Department of Veterans Affairs, 2021a). They also encouraged Veterans to request an appointment to address questions about continuing or discontinuing use of their device while awaiting replacement.

Upon attending the appointment, Veterans could ask questions; provide information about their health and use and condition of the device; receive information about risks; and express preferences about how to proceed. Based on these inputs and the provider's assessment of clinical need, the provider could then collaboratively formulate a treatment plan with the Veteran and document it in the electronic health record. The treatment plan would balance, for each Veteran awaiting a replacement device, the risks and benefits of continuing to use their devices vs. initiating other therapy such as surgical treatments, oral appliance therapy, weight loss, nasal end expiratory pressure devices, or positional therapy.

#### 3.4.3. Stratifying Veterans by risk of harm from recalled devices

In order to maintain a transparent and consistent approach to counseling Veterans, the recall response team stratified Veterans into tiers of clinical need based on risk of harm from the recalled devices. Sleep medicine leaders reviewed the manufacturer's analysis and concluded that the highest risk Veterans, denoted Tier 1, were those with comorbidities and active symptoms temporally associated with use of a recalled device; Veterans with an older device (issued more than 5 years ago); Veterans who had used an UV/ozone cleaner known to accelerate foam degradation; or Veterans with devices exhibiting visible particulate matter. VHA determined that such Veterans should be highest priority for obtaining a replacement device. The next risk tier (Tier 2) included Veterans with moderate to severe sleep apnea and known comorbidities but no active symptoms. Next after these (in Tier 3) were Veterans with mild sleep apnea with no comorbidities and no active symptoms. To help clinicians understand the process, the recall response team created a visual aid in July 2021 to explain the tier system and how to identify which tier patients were in. The visual aid included alternate therapy suggestions for those in Tiers 2 and 3 that would have to wait for a replacement device.

#### 3.4.4. Helping providers guide Veterans with a clinical note template

The recall response team propagated VHA's approach to counseling through a clinical note template in the electronic health record system. Sleep providers could then rely on the structured template to guide their counseling and documentation. In consultation with other VHA sleep medicine experts, author KS drafted the contents of the template. VHA's human factors review team assessed the note, entitled “CPAP, BiPAP, HMV Philips Respironics Recall Note.” PAP is the acronym used for Positive Airway Pressure, with C denoting Continuous and Bi denoting Bilevel (BiPAP is a registered trademark of Philips RS North America LLC). HMV is the acronym for Home Mechanical Ventilator.

After human factors review, Clinical Application Coordinators from each facility programmed the note into VHA's electronic health record. Clinicians accessed the note through a shared template available to place in a new note or one started as part of a clinical visit. Once in the template, they could select a pathway for evaluating a Veteran with a recalled device.

This pathway used branching logic in assessing medical history, comorbidities, sleep disordered breathing severity; use and age of the recalled device; whether UV/ozone cleaners were ever used (a known risk factor for foam degradation); and presence and duration of symptoms that could be related to foam degradation. The template prompted the clinician to discuss risks and benefits of discontinuing vs. continuing use of a recalled device as follows:

Overall risks associated with continued device use are very low. However, using an older device (system one) or one which has been exposed to ozone/UV cleaners increases the likelihood of degradation of the foam. Use of any ozone/UV cleaners should stop immediately. Particulate matter and chemicals from the foam have the potential to cause toxic and carcinogenic affects, although no cancers are known to be linked to the device at this time.

The risks of discontinuing therapy entirely were discussed. Risk of untreated sleep apnea is increased in those with severe sleep disordered breathing, excessive daytime sleepiness, those with chronic respiratory failure and hypoventilation syndromes (advanced pulmonary disease, neuromuscular disease, and obesity hypoventilation), and those with cardiovascular and cerebrovascular disease. Risk is also increased in Veterans with mood disorders and PTSD where treatment of sleep apnea has resulted in clinical improvement and cessation of treatment may lead to clinical worsening.

The first pathway concluded with the clinician documenting the treatment decision:

Together with the veteran, the decision was made [among mutually exclusive choices] to:

Continue treatment with the current PAP device until a replacement is provided by Philips Respironics. The patient understands there is no guaranteed timeline for this process, which is beyond the control of VA, and it may be several months or even more than a year before a new device may be available to the Veteran (Tier 1b, Tier 2).

OR

Replace current Philips Respironics device with device unaffected by the recall from VA (Tier 1a only, if devices are available).

OR

Discontinue PAP therapy altogether and pursue alternative strategies if applicable (Tier 2, Tier 3).

To train clinicians in the use of the clinical note template, the recall response team presented an interactive training webinar open to all VHA sleep providers on the launch date for the template, September 21, 2021. This initial training occurred during the weekly recall office hours, described in the Communications section above. This meeting series included 793 VHA sleep providers as invitees. The recall response team provided additional coaching during subsequent office hours, and in the chat channel. The team also made available the screen recordings of all office hours, along with associated files such as instructions with screenshots.

## 4. Discussion

### 4.1. Strategic plan lessons learned

#### 4.1.1. Assembling a cross-functional recall response team

Author JF cited prior working relationships as a key facilitator for rapidly assembling a multi-disciplinary recall response team. Members of the recall response team also cited their mutual familiarity as a success factor in promoting a rapid response and collegial collaboration under crisis conditions. Incorporating communications professionals as part of the response team from day 1 was key to building familiarity with the issues and delivering appropriate Veteran-centered messages and information throughout the response.

#### 4.1.2. Deferring to clinician assessment of Veteran clinical need

VHA's national leaders in the areas of patient safety and communications readily agreed that VHA's response should be Veteran-centered and led by clinicians. This was consistent with VHA's overall core values of Integrity, Commitment, Advocacy, Respect, and Excellence, as articulated in its strategic plan. VHA's organizational alignment behind clinician leaders facilitated its Veteran-centered organizational response. One lesson here is that core values and strategic plans can provide a common language and scaffolding for teams to rely on in a crisis, so that everyone remains focused on advancing the long-term interests of key stakeholders.

#### 4.1.3. Communicating transparently about the recall

The recall response team felt that their multi-channel communications campaign established a precedent to guide future responses to recalls and other crises. The multi-pronged strategy met the varied information processing needs of the audience. Clinicians could attend weekly and then biweekly online meetings, where they reviewed auditory and visual information presented by experts, and could ask questions and get responses live. Or, they could access information asynchronously, reviewing the slides and recordings presented at online meetings after the fact; and explore reference documents in a centralized library. Similarly, Veterans could review summary information received *via* letter or email; or pursue more detailed explanation in public websites or by scheduling an appointment with a provider.

VHA's prior investments in information technology allowing for centralized online publication facilitated communication from the recall response team to the field. VHA also more recently invested in technology for large-group online meetings where recall team members could synchronously present and answer audience questions. The same technology platform also allowed for asynchronous “chat” based communication among members of the same distribution list. This technology facilitated interactive communication between the recall response team and other stakeholders.

#### 4.1.4. Allocating scarce resources based on an explicit ethical framework

Clinicians leading the response called upon VHA's National Center for Ethics in Health as a source of expertise and guidance. In doing so, recall response team members cited the prior guidance of this Center in scarce resource allocation frameworks during COVID-19 as well as for hepatitis C medication. Having an ethical framework helped the organization align itself behind a consistent, Veteran-centered approach.

#### 4.1.5. Inviting Veterans to schedule appointments with sleep providers

Clinicians felt that the ethical resource allocation framework, with its commitment to stratifying risks, required clinicians to assess some of the risks directly from Veterans. This in turn implied inviting Veterans to interact with sleep providers. While chart review would have been more efficient, it would not have been as effective or Veteran-centered. Here again, VHA's core values guided the implementation of a strategic decision to allocate scarce devices according to Veteran need.

### 4.2. Service plan lessons learned

The recall response team was able to quickly frame the service plan in terms of revisiting prior PAP therapy decisions. VA's strategic plan facilitated this reasoning because VA has adopted a user-centered design perspective. In design thinking, service delivery plans revolve around the customer's journey. VA Strategy 1.1.3 states that “VA will expand the use of the Veteran journey maps” and this directive encouraged the recall response team to think about the recall as one step in the Veteran's overall journey.

### 4.3. Operational plan lessons learned

#### 4.3.1. Identifying Veterans affected by the recall

Recall response team members identified VHA's investment in the National Prosthetic Patient Database as a key facilitator in identifying patients with affected devices and communicating with patients about the need to schedule appointments with sleep providers.

#### 4.3.2. Informing Veterans and inviting them to schedule appointments with VHA sleep providers

Thanks to its centralized communications infrastructure, VA was able to send emails to Veterans alerting them to their recall response options, and inviting them to schedule visits with providers. Based on technological tracking of which Veterans had opened the emails, VA was able to send follow-up emails and then letters by mail.

#### 4.3.3. Stratifying Veterans by risk of harm

The manufacturer's analysis was helpful in guiding the recall response team's stratification of Veteran risks. The manufacturer's indications could in principle be observed, inferred, or reported: age of device; use of ozone cleaners; and visibility of particulate matter. This facilitated communication and consistent implementation of VA' risk tiering process.

#### 4.3.4. Helping providers guide Veterans with a clinical note template

As a facilitator of Veteran engagement, recall response team members cited VHA's ability to deploy a structured note template in the electronic health record system. Each health care system in the VHA network has Clinical Application Coordinators who can update the electronic health record with new templates. This meant VHA could distribute nationally the recall response team's algorithm with branching logic and scripted prompts. Clinicians could therefore apply the logic and prompts consistently, for equitable administration of the decision making process.

## 5. Strengths, limitations, and conclusions

One strength of this report is that the recall response team used program monitoring and reflected critically on VHA's response to the recall in real time. The team worked with an evaluator with the intention of engaging in course correction, and in order to summarize summarizing and publish lessons learned for the benefit of future audiences. Therefore, we have summarized the issues encountered with high fidelity to the real-time unfolding of the recall.

The existence and composition of the recall response team also constitutes a strength. Team members came from diverse organizational units. All team members reflected VHA's commitment to being a high reliability organization in the way that they deferred to expertise. Thus, the subject matter experts from sleep medicine led the recall and, because they were closest to the customer (Veteran), kept the response Veteran-centered.

One limitation of this report is that it presents only one case, outlining the response at one system, and without evaluating whether the response was successful or how it compared to other recall responses. One reason for this is that the recall is not yet complete: the recall coincided with a worldwide pandemic that interrupted and disrupted supply chains. This meant that the availability of raw materials for manufacturing replacement devices varied, and so the quantity and pace of remediation varied over time, and was still under way as we formulated our report. We have reported on our program processes because of the importance of initial responses to a crisis like a recall. While we monitored and report on our organization's internal processes, evaluating the impact of our response was outside of our scope. Another limitation is that we are presenting only VHA's organizational response to the recall. This omits other important perspectives that deserved to be shared, including those of Veterans, and the manufacturer.

Our overall conclusions are that our team captured useful lessons by using program monitoring techniques to reflect critically in real time on VHA's organizational processes related to the recall. Real-time program monitoring contributed to improved clarity of communications and therefore alignment of key stakeholders in the field.

We conclude with recommendations for other health systems facing recalls. Our experience suggests strategies to be considered before, during, and after any recall.

Before a recall, consider anticipating the possibility of a recall for medical devices in use within a system. Health systems should consider stockpiling a reserve of medical devices for use in case of emergencies that would threaten the lives of their patients. Because recalls are so disruptive to normal healthcare operations, purchasers could write terms into procurement contracts whereby manufacturers would be held accountable for maintaining supply to highest need patients. Analogous service level agreements, with incentives and penalties, are common in the provision of, say, critical information technology services (Wazir et al., 2016). Information technology providers have learned to build redundancy into their operations, anticipating disruptions such as earthquakes or sabotage, so that they can restore service quickly and guarantee a certain level of uptime. The same could be true of medical device providers.

Another pre-recall recommendation is to have clearly articulated core values that the organization can rely on to guide decisions during a recall, or indeed any crisis response. As management scientists have pointed out, a clear strategic plan, including the organizational purpose, mission, and core values will serve as a compass for organizational pathfinding, and can help organizations make and explain difficult trade-offs (Collins and Porras, 1994).

Organizations can also designate, in advance of any specific recall, a cross-functional recall response team. The Mayo Clinic report emphasizes the importance of having a team and a scaffolding in place for recall response. The Mayo Clinic scaffolding consists of three lessons learned from prior recalls. We propose expanding this scaffolding to include three dimensions of program monitoring: a strategic plan, a service plan, and an operational plan.

During a recall, we recommend that organizations consider how a device was first adopted or distributed. Returning to that workflow can ensure that all original stakeholders also participate in the remediation of the recall. Assuring that stakeholders have a voice in the resolution of the recall can increase the quality of the response and reduce conflict. Including the original stakeholders in creating solutions is also consistent with a high reliability principle of deferring to expertise (Merchant et al., 2022).

One issue that will arise during recalls is the type and level of ascertainment required, for example, who will be responsible for determining whether a device qualifies as recalled and whether it is eligible for replacement or remediation. Who will bear the cost of this ascertainment? Who will be accountable if too few devices are replaced because the standard for determining eligibility created barriers to front-line participation in the recall? Who will bear the cost if organizations err on the side of broadly replacing or remediating too many rather than too few devices? These are issues that organizations can also anticipate and write into procurement contracts.

During a recall, organizations should consider monitoring the process of the recall in order to reflect critically, in real time, and engage in course correction. This will naturally flow into the post-recall period, when organizations should document and share lessons learned so that institutional knowledge improves regarding recall response.

Two other organizations, the Cleveland Clinic and Mayo Clinic, have also published reflections and lessons learned. Comparing their lessons learned with ours, we note some similarities and differences. All three organizations cite the use of structured decision algorithms, and of electronic health records and patient portals to facilitate communication about the recall and guidance to patients. Smart phrases (keyboard shortcuts that insert blocks of text) represent an opportunity to standardize communication on a mass scale, and electronic health record systems generally support this functionality. Organizations can also implement decision algorithms, sometimes through surveys with branching logic that guide the user through assessment steps toward a disposition based on dynamic inputs. Mayo, Cleveland Clinic, and VHA quickly deployed algorithms used by providers; during COVID-19, organizations developed and deployed similar branching logic algorithms for patients to self-administer in screening for symptoms and obtaining guidance about testing, tracing, treatment, and vaccination (Judson et al., 2020; Meer et al., 2021).

In summary, the period immediately after a critical incident such as this recall is the time to apply lessons learned. We must all recognize that the aftermath of one recall is the prequel to the next. Health care systems that distribute devices or support patients using devices should form recall response teams as part of their emergency preparedness. These teams should develop strategic, service, and operational plans to prevent, mitigate, and recover from device recalls that will inevitably occur in the future. The next research frontier in this domain is to evaluate the effectiveness of using strategic, service, and operational plans in responding to recalls. In addition, future studies should compare the effectiveness of this approach to other approaches described in the literature. In this way, researchers can further advance the science of responding to recalls.

## Data availability statement

The raw data supporting the conclusions of this article will be made available by the authors, without undue reservation.

## Author contributions

JB outlined and drafted the manuscript. All authors participated in writing or revising the manuscript. All authors contributed to the article and approved the submitted version.
